# Salivary DNA methylation panel to diagnose HPV-positive and HPV-negative head and neck cancers

**DOI:** 10.1186/s12885-016-2785-0

**Published:** 2016-09-23

**Authors:** Yenkai Lim, Yunxia Wan, Dimitrios Vagenas, Dmitry A. Ovchinnikov, Chris F. L. Perry, Melissa J. Davis, Chamindie Punyadeera

**Affiliations:** 1 The School of Biomedical Sciences, Institute of Health and Biomedical Innovation, Queensland University of Technology, GPO Box 2434, 60 Musk Avenue, Kelvin Grove, Brisbane, QLD 4059 Australia; 2 Australian Institute for Bioengineering and Nanotechnology, University of Queensland, St Lucia, Brisbane, QLD 4072 Australia; 3 Department of Otolaryngology, Princess Alexandra Hospital, 199 Ipswich Road, Woolloongabba, Brisbane, QLD 4102 Australia; 4 School of Medicine, University of Queensland, 288 Herston Road, Herston, Brisbane, QLD 4006 Australia; 5 Department of Biomedical Engineering, University of Melbourne, Parkville, Melbourne, VIC 3010 Australia

**Keywords:** Saliva, Tumour-suppressor genes, Human Papillomavirus, Head and neck cancers, DNA methylation, Epigenetics biomarkers, Cross-fold validation and early detection

## Abstract

**Background:**

Head and neck squamous cell carcinoma (HNSCC) is a heterogeneous group of tumours with a typical 5 year survival rate of <40 %. DNA methylation in tumour-suppressor genes often occurs at an early stage of tumorigenesis, hence DNA methylation can be used as an early tumour biomarker. Saliva is an ideal diagnostic medium to detect early HNSCC tumour activities due to its proximity to tumour site, non-invasiveness and ease of sampling. We test the hypothesis that the surveillance of DNA methylation in five tumour-suppressor genes (*RASSF1α*, *p16* ^*INK4a*^, *TIMP3*, *PCQAP*/*MED15*) will allow us to diagnose HNSCC patients from a normal healthy control group as well as to discriminate between Human Papillomavirus (HPV)-positive and HPV-negative patients.

**Methods:**

Methylation-specific PCR (MSP) was used to determine the methylation levels of *RASSF1α*, *p16* ^*INK4a*^, *TIMP3* and *PCQAP*/*MED15* in DNA isolated from saliva. Statistical analysis was carried out using non-parametric Mann-Whitney’s *U*-test for individually methylated genes. A logistic regression analysis was carried out to determine the assay sensitivity when combing the five genes. Further, a five-fold cross-validation with a bootstrap procedure was carried out to determine how well the panel will perform in a real clinical scenario.

**Results:**

Salivary DNA methylation levels were not affected by age. Salivary DNA methylation levels for *RASSF1α*, *p16* ^*INK4a*^, *TIMP3* and *PCQAP*/*MED15* were higher in HPV-negative HNSCC patients (*n* = 88) compared with a normal healthy control group (*n* = 122) (sensitivity of 71 % and specificity of 80 %). Conversely, DNA methylation levels for these genes were lower in HPV-positive HNSCC patients (*n* = 45) compared with a normal healthy control group (sensitivity of 80 % and specificity of 74 %), consistent with the proposed aetiology of HPV-positive HNSCCs.

**Conclusions:**

Salivary DNA tumour-suppressor methylation gene panel has the potential to detect early-stage tumours in HPV-negative HNSCC patients. HPV infection was found to deregulate the methylation levels in HPV-positive HNSCC patients. Large-scale double-blinded clinical trials are crucial before this panel can potentially be integrated into a clinical setting.

**Electronic supplementary material:**

The online version of this article (doi:10.1186/s12885-016-2785-0) contains supplementary material, which is available to authorized users.

## Background

Head and neck squamous cell carcinomas (HNSCCs) encompasses tumours within the oral cavity, pharynx, larynx, paranasal sinuses, nasal cavity and salivary glands, and are some of the most aggressive cancer types [1, 2]. Main risk factors for HNSCC include smoking, alcohol consumption, betel quid chewing, and Human Papillomavirus (HPV, mainly HPV-16 and HPV-18) infections [2–4]. HPV-positive and HPV-negative cancers are biologically and clinically different and as such require different treatment and clinical management [5].

HNSCC is the sixth most common cancer worldwide with ~780,000 new cases diagnosed each year [6]. The incidence of HNSCC in developed countries has decreased over the past 20 years, which is largely attributed to a reduction in smoking and alcohol consumption [4]. However, the incidence of HPV-positive HNSCC is on the rise and accounts for 30 to 50 % of all HNSCCs [4, 6]. HPV-positive HNSCC patients have cancers that are almost exclusively located in the oropharynx [7–10]. HPV-positive HNSCC patients are often young with a higher socioeconomic status and typically non-smokers [7–10]. The five-year survival rates for HPV-negative HNSCC when diagnosed early is 80 % compared with only 15 % for the advanced stage cancers [11–13].

Currently, diagnosis relies on the direct examination of the head and neck regions and is usually made after clinical presentation of symptoms and involves biopsy to confirm diagnosis. The HPV status of a patient is determined by p16^INK4a^ immunohistochemistry (IHC) staining on tumour tissue samples and histological classification using the tumour-node-metastasis (TNM) [14–16]. Direct examination is highly-subjective and becomes problematic when tumours are too small to be visualised, or are hidden in obscure areas such as the tonsillar crypts or within the pits and crevices in the lingual tonsils of the tongue base. This would then likely require techniques such as nasendoscopy or examination under anaesthesia to locate the tumour and both require biopsy for confirmation. These issues may commonly result in misdiagnosis [17]. The direct contact between saliva and oral cavity lesions make the measurement of the tumour markers in saliva an attractive alternative to serum and tumour tissue biopsy testings [6, 18–21]. Saliva is now championed as the diagnostic fluid of the future over blood and urine as saliva testing is easy, inexpensive, safe, and non-invasive [19, 22–25].

Gene-specific DNA methylation, especially in tumour-suppressor genes, is recognized as a contributor to the regulation of gene expression and phenotypic heterogeneity in HNSCC [26, 27]. The DNA promoter methylation analysis in saliva samples collected from HNSCC patients have previously been shown to demonstrate clinical utility [6, 25, 28]. The most commonly used method for the detection of DNA methylation analysis in tissue and body fluids is the methylation-specific PCRs (MSPs) [29]. MSP analysis is highly sensitive and does not require expensive laboratory equipment and is therefore economical compared to other quantitative DNA methylation analysis such as pyrosequencing and real-time quantitate MSP [30, 31]. In addition, MSP is able to provide a time-efficient and direct DNA methylation status analysis, making it convenient for large-scale sample screening [30, 31]. The ability to relatively quantify DNA methylation signatures allows the delineation of clinically meaningful threshold values to discriminate a patient cohort from a control cohort.

We hypothesise that by analysing DNA methylation of tumour-suppressor genes in saliva; we can detect early tumour activities as well as to differentially diagnose HNSCC patients. Our study objectives are two-fold: (i) firstly, to investigate the early diagnostic potential of the salivary DNA methylation panel (*RASSF1α*, *p16* ^*INK4a*^, *TIMP3*, *PCQAP* 5′ and *PCQAP* 3′) (ii) secondly, to determine whether this panel is able to discriminate between HPV-negative and HPV-positive HNSCC patients. We selected this panel as we have previously published individual DNA methylation levels in saliva collected from HNSCC patient and controls except for *TIMP3*. From our previously published work, we were able to discriminate normal healthy controls from HNSCC patients using these individual DNA methylation levels [6, 25]. In this study, we have combined the DNA methylation levels for all of the five tumour-suppressor genes as a panel to increase the sensitivity and specificity when discriminating normal healthy controls from HNSCC patients. Our salivary DNA methylation panel is able to detect HPV-negative HNSCC patients from a normal healthy control group with a sensitivity of 71 % and specificity of 80 %. In contrast, the DNA methylation levels were lower in saliva collected from HPV-positive HNSCC patients compared with normal healthy controls (sensitivity of 80 % and specificity of 74 %). It is important to conduct a multi-centre clinical trial before this panel can be implemented in a clinical setting.

## Methods

### Study design

This study is approved by the University of Queensland (HREC no.: 2014000679) and Queensland University of Technology (HREC no.: 1400000617) Medical Ethical Institutional Boards and the Princess Alexandra Hospital’s (PAH) Ethics Review Board (HREC no.: HREC/12/QPAH/381). We have recruited normal healthy controls, both smokers and non-smokers (*n* = 122) and HNSCC patients (*n* = 133). HNSCC patient cohort consisted of HPV-negative and HPV-positive patients. The Table 1 presents the demographic and clinical characteristics of our study cohort.

**Table 1:** The demographic characteristics of the study cohort (*n* = 255)

Explanatory variables	Controls	Patients	
		HPV -ve	HPV + ve
	*n* = 122 (47.8 %)	*n* = 88 (34.5 %)	*n* = 45 (17.7 %)
Demographics			
Gender			
Male	54 (44.3)	67 (76.1)	42 (93.3)
Female	68 (55.7)	21 (23.9)	3 (6.7)
Age			
< 50	66 (54.1)	8 (8.6)	7 (15.6)
50–59	42 (34.4)	25 (26.9)	17 (37.8)
> 60	14 (11.5)	60 (64.5)	21 (46.7)
Race and ethnicity			
Caucasian	107 (87.7)	86 (97.7)	43 (95.6)
Asian	8 (6.6)	0 (0)	0 (0)
Other	7 (5.7)	2 (2.3)	2 (4.4)
Smoking			
Pack/day smoked (cigarettes, cigar or pipe)			
Non-smoker	86 (70.5)	14 (15.1)	12 (26.7)
Ex-smoker	7 (5.8)	40 (43.0)	23 (51.1)
1 to 19	17 (13.9)	27 (29.0)	8 (17.8)
> 20	6 (4.9)	6 (6.5)	2 (4.4)
Unknown	6 (4.9)	1 (1.1)	0 (0)
Drinking			
No. Of years drank >15 drinks per week			
Non-drinker	9 (7.4)	2 (2.3)	5 (11.1)
Ex-drinker	0 (0)	3 (3.4)	4 (8.9)
1 to 14	31 (25.4)	6 (6.8)	15 (33.3)
> 15	3 (2.4)	11 (12.5)	7 (15.6)
Unknown	79 (64.8)	66 (75.0)	14 (31.1)
Tumour characteristics			
AJCC TNM stage			
Stage 0		0 (0)	0 (0)
Stage I		17 (19.3)	2 (4.4)
Stage II		15 (17.0)	2 (4.4)
Stage III		10 (11.4)	7 (15.6)
Stage IVa		23 (26.1)	26 (57.8)
Stage IVb		2 (2.3)	4 (8.9)
Stage IVc		1 (1.1)	0 (0)
Unknown		20 (22.7)	4 (8.9)
Tumour anatomic site			
Oral cavity		67 (76.1)	4 (8.9)
Oropharynx		11 (12.5)	39 (86.7)
Hypopharynx		2 (2.3)	0 (0)
Larynx		6 (6.8)	1 (2.2)
Neck		2 (2.3)	1 (2.2)

### Determination of HPV-16 status in tumour samples

We obtained a pathology report for each patient which contained tumour staging information, histopathological grading and HPV-16 status. HPV-16 status was determined by staining for p16^INK4a^ in tumour tissue section using IHC (CINtec® Histology Kit, Roche MTM Laboratories, Heidelberg, Germany) according to the manufacturer’s protocol [32]. p16 ^INK4a^ IHC was evaluated by trained pathologists [32]. The determination of HPV-16 status at the PAH is restricted to patients with cancers in the oropharynx because of the low prevalence of HPV-16 among non-oropharynx sites [9]. Therefore, *p16* ^*INK4a*^ IHC is not requested by the treating clinician when tumours are outside of the oropharyngeal area.

### Saliva sample collection and processing

In the clinic, volunteers were asked to refrain from eating and drinking for an hour prior to donating saliva samples. The volunteers were asked to sit in a comfortable position and were asked to rinse their mouths with water to remove food debris. They were then asked to pool saliva in the mouth and expectorate directly into a 50 mL Falcon tube. Saliva samples were transported from the hospital to the laboratory on dry ice. Samples were centrifuged at 1500 × g for 10 min at 4 °C, separating cellular pellet from cell-free salivary supernatant. Cellular pellet was used to isolate DNA, which was subsequently subjected to bisulfite conversion.

### DNA extraction and bisulfite conversion from saliva samples

The Epitect® Plus DNA Bisulfite Kit (Cat. No. 59124, Qiagen, Duesseldorf, Germany) was used to extract and bisulfite-convert DNA from salivary cellular pellet according to the manufacturer’s protocol. An additional 10 min of incubation time was adapted due to a change in elution volume of 17 μL instead of 15 μL. Purity and quantity of the converted DNA samples were measured with a Nano Drop ND-1000 spectrophotometer (Thermo Fisher Scientific, Waltham, Massachusetts, USA).

### Methylation-specific PCR assays

The MSP primer pairs (*RASSF1a*, *p16* ^*INK4a*^, *TIMP3*) used in this study has been extensively validated in other studies, except for *MED15/PCQAP* [6, 25]. *MED15*/*PCQAP* novel CpG sites were identified by our group and we have previously confirmed the specificity of amplicons using the MSP primer pairs and we have also verified the PCR amplicon sequence (Additional file 1: Figure S1) [25]. The primer specificities for *RASSF1a* and *p16* ^*INK4a*^ were confirmed by Divine et al., 2006 using the denaturing high performance liquid chromatography (DHPLC) [6, 33]. Similarly, *TIMP3* MSP primer pairs was initially used in a MethyLight assay by Eads et al. in 2001 and later modified by Righini et al. to be compatible with a MSP assay [34, 35].

To determine the specificity of the MSP primers, all MSP primers (both methylation and unmethylation) were tested using bisulfite unconverted DNA samples and was found not to amplify. This proves the specificity of the primer pairs used in this study. Unmethylation PCRs were used as a normaliser for methylation PCRs. Samples without unmethylation bands were either discarded from the analysis or repeated. Bisulfite-treated methylated HeLa cell line DNA (Cat. No.4007s, New England Biolabs, Ipswich, Massachusetts, USA) was used as a positive control while DNase/RNase-free distilled water (blank) was used as a negative control for the MSP assays.

**Fig. 1:**
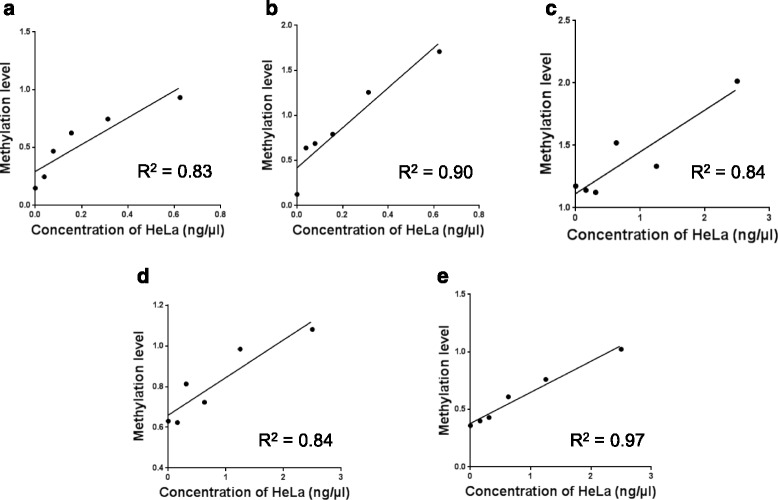
A six-point standard curve spiking of positive cell line, HeLa in oral adenosquamous cell carcinoma, CAL27 of **a** *RASSF1α*, **b** *p16* ^*INK4a*^, **c** *TIMP3*, **d** *PCQAP* 5′ and **e** *PCQAP* 3′

The quantitative nature and efficiency of conventional MSP was established by using bisulfite-treated methylated HeLa cells at varying amounts. In brief, HeLa cells were spiked in oral adenosquamous carcinoma cell line, (CAL27) in a six-point serial dilution format to generate a standard curve using the ratio of methylation to unmethylation PCR reactions (Fig. 1) [36]. Our results clearly demonstrate that the conventional MSP is a reliable way to relatively quantify methylation levels (MSP efficiencies of >0.8) (Fig. 1).

*RASSF1α* and *p16* ^*INK4a*^ were amplified using nested MSP. Nested MSP primer sets for both stage-1 (nested, methylation-insensitive stage) and 2 (methylation-sensitive stage) are presented in Table 2 [6]. Briefly, stage-1 PCR amplification for *RASSF1α* and *p16* ^*INK4a*^ was carried out using 1 μM of the appropriate nested primer sets, 6.25 μL of EmeraldAmp® MAX HS PCR Master Mix (TaKaRa Bio Inc., Otsu, Shiga, Japan) and 1.25 ng and 20 ng of DNA template respectively. The total reaction volume of 12.5 μL was subjected to PCR amplification using the following conditions: initial denaturing stage at 94 °C for two minutes, followed by 30 cycles of 15 s at 94 °C, 15 s at 60 °C and 15 s at 72 °C. In stage-2, two corresponding sets of methylated and unmethylated primers for each gene were used. The amplification cycling conditions included: initial denaturing stage at 94 °C for 2 min, followed by 5 cycles of 15 s at 94 °C, 15 s at 62 °C and 15 s at 72 °C with three repeats of decreasing annealing temperature (64, 62 and 60 °C in that order) before extension stage at 72 °C for 5 min. Stage-2 PCRs used 1 μL of stage-1 product as DNA template.

**Table 2:** Methylation specific PCR primer sequences

Gene	Nucleotide sequence	PCR product size, base pair (bp)
Methylation-independent primer sequences (nested)		
*RASSF1α*	Forward: 5′-GGAGGGAAGGAAGGGTAAGG-3′	260
	Reverse: 5′-CAACTCAATAAACTCAAACTCCC-3′	
*p16* ^*INK4a*^	Forward: 5′-GAGGAAGAAAGAGGAGGGGTTG-3′	274
	Reverse: 5′-ACAAACCCTCTACCCACCTAAATC-3′	
Methylated allele-specific primer sequences		
*RASSF1α*	Forward: 5′-GGGGGTTTTGCGAGAGCGC-3′	203
	Reverse: 5′-CCCGATTAAACCCGTACTTCG-3′	
*p16* ^*INK4a*^	Forward: 5′-GAGGGTGGGGCGGATCGC-3′	143
	Reverse: 5′-GACCCCGAACCGCGACCG-3′	
*TIMP3*	Forward: 5′-GCGTCGGAGGTTAAGGTTGTT-3′	116
	Reverse: 5′-CTCTCCAAAATTACCGTACGCG-3′	
*PCQAP* 5′	Forward: 5′-GTTTTGTGATTGAGGYGGCGGC -3′	167
	Reverse: 5′-AAAAATCCCACAATCCAACCC -3′	
*PCQAP* 3′	Forward: 5′-GATATGGGTGGTGGGAGTTGGG -3′	172
	Reverse: 5′- AATCAGACCCTAACCTCGCCCG -3′	
Unmethylated allele-specific primer sequences		
*RASSF1α*	Forward: 5′-GGTTTTGTGAGAGTGTGTTTAG-3′	172
	Reverse: 5′-ACACTAACAAACACAAACCAAAC-3′	
*p16* ^*INK4a*^	Forward: 5′-TTATTAGAGGGTGGGGTGGATTGT-3′	145
	Reverse: 5′-CAACCCCAAACCACAACCATAA-3′	
*TIMP3*	Forward: 5′-TGTGTTGGAGGTTAAGGTTGTTTT-3′	122
	Reverse: 5′-ACTCTCCAAAATTACCATACACACC-3′	
*PCQAP* 5′	Forward: 5′-GTTTTGTGATTGAGGYGGTGGT -3′	167
	Reverse: 5′-AAAAATCCCACAATCCAACCC -3′	
*PCQAP* 3′	Forward: 5′- TGATTAATTTAGATTGGGTTTAGAGAA -3′	158
	Reverse: 5′- CCAACTCCAAATCCCCTCTCTAT -3′	

For *TIMP3*, unique methylated and unmethylated primer sets for each gene was used to target their corresponding CpG-methylation sites (Table 2) [34]. The PCR reaction consisted of 5 μL of EmeraldAmp® MAX HS PCR Master Mix and 0.8 μM of their respective primer sets, in 10 μL final reaction volume. Total DNA template ratio of 20:1 was used for the methylated reaction and unmethylated reaction respectively. The PCR amplification consisted of initial denaturing stage at 95 °C for 5 min, followed by 40 cycles of 15 s at 94 °C, 15 s at 54 °C and 15 s at 72 °C before summing up with elongation stage at 72 °C for 4 min.

Similar to *TIMP3*, *PCQAP* (Table 2) also required two separate setup conditions for the methylated and unmethylated reactions under the same cycling condition. Both methylated and unmethylatd reactions consisted of 6.25 μL of EmeraldAmp® MAX HS PCR Master Mix and 1 μM of their respective primer sets. In terms of DNA template concentrations, ratio of 25:1 was used for the methylated reactions and unmethylated reactions respectively. The PCR amplification consisted of initial denaturing stage at 95 °C for 3 min, followed by 35 cycles of 30 s at 94 °C, 30 s at 62.5 °C and 1 min at 72 °C before summing up with elongation stage at 72 °C for 5 min. *PCQAP* MSP reactions required an addition of 5 % DMSO and 0.1 μg/mL BSA to minimise the presence of unspecific bands caused by secondary DNA structures [25].

### Gel electrophoresis and densitometry analysis

MSP analysis was carried out by running 5 μL PCR amplicon products on 2 % agarose gel. The gels were scanned on Fusion SL (Vilber Lourmat, Marne la Vallee, France) and visualized using ImageJ software (National Institutes of Health, Bethesda, Maryland, USA). In order to quantify the ratio between methylated and unmethylated bands, samples with saturated bands were re-run with a lower concentration ratio of DNA template for both methylated and unmethylated PCRs.

The methylated and unmethylated band intensities were quantified using ImageJ software and the ratio between methylated to unmethylated was calculated for each sample using Microsoft Excel (Microsoft Corporation, Redmond, Washington, USA). A standard rectangular-frame was estimated according to the size of the smallest band in a given gel. Consequently, the same rectangular-frame was used to measure the intensity of each band within the same gel to provide consistency. The measurement was set at integrated density to calculate the intensity value of the band based on the amount of amplicon present. All quantifications were carried out by two independent researchers to minimise observational errors.

### Statistical analysis

The statistical analysis was carried out by using GraphPad Prism (GraphPad Software, Inc, San Diego, California, USA) and R (R.D.C. Team, Vienna, Austria). The methylation levels were not normally distributed and therefore a non-parametric test (Mann-Whitney *U* test) was used when comparing the data generated using normal healthy controls with HPV-negative and HPV-positive HNSCC patients respectively. In addition, Spearman’s rank correlation test was used to determine the correlation between patients’ age and methylation level given that age is a continuous variable.

The overarching aim of this study is to evaluate the diagnostic potential of the combined five tumour-suppressor genes in a panel and as such, the sensitivity and specificity were estimated. For this purpose, the ‘Epi’ package was used in R [37]. The patient status is used as the outcome variable and the methylation level for each gene is used as the explanatory variables in a multivariable logistic regression (Carstensen’s multivariate ROC curve). Predicted scores are then produced for each patient using the estimated regression model and different cut-off values of this predicted score are used for classifying samples into patients or controls. A known issue in this case is that the predicted classification of the samples is optimal since the same sample that has been used for creating the predicting model and for validating it. One good solution to address this type of issue is known as cross validation, the idea of which that proportion of the sample is used for creating the predictive model, and the remaining samples are used for validating the model [38–40]. In this case, a version of five-fold cross-validation was used. This is crucial to see how well the panel translates into clinical diagnosis. To make best use of our data, a bootstrap procedure was also incorporated [38–40]. With this statistical method, random samples are created by sampling with replacement from the original sample. The advantage of this technique is that the confidence intervals produced are more realistic compared to the parametric, asymptotic ones. Furthermore, this was done in a stratified manner; classifying on patient status in order to retained the original samples’ characteristics. Therefore, this procedure could be called a stratified bootstrap ROC with cross-validation. A custom written code was used to implement this in R using the above function from R. The program was ultimately run for 5000 times to include all possible combinations of predictive model available. The maximum sum of sensitivity and specificity was used to determine the best cut-off point for the panel.

### TCGA data portal

To investigate the tumour methylation status of the five genes, we downloaded The Cancer Genome Atlas (TCGA) data for HNSCC tumours and normal tissues (https://tcga-data.nci.nih.gov/tcga/). HPV status annotation was available for 268 tumours profiled by Tang et al., (DOI:10.1038/ncomms3513; Additional file 2: Table S1) [41]. Tumours were grouped as HPV-positive HNSCC (*n* = 44), HPV-negative HNSCC (*n* = 223), or normal tissue samples (*n* = 50). Our approach was to select probes that overlapped within the CpG sites flanking our primer pairs used in our MSP assays (Additional file 3: Figure S2). Probes for *RASSF1α*, *TIMP3* and *PCQAP* were extracted and the DNA methylation values for these three groups were plotted in R. However, there were no probes that overlapped or positioned adjacent to the CpG methylation sites interrogated by our *p16* ^*INK4a*^ MSP assays. As such, we were unable to present TCGA data for *p16* ^*INK4a*^.

## Results

### Population characteristics

The mean age for normal healthy controls was 50 years (SD: 8.4 years), and consisted of 44.3 % men and 55.7 % women (Table 1). The mean age for HNSCC patients was 64 years (SD: 12.2 years), and consisted of 82.0 % men and 18.0 % women (Table 1). Cancer sites were mostly of oropharyngeal and oral cavity (53.4 and 37.6 % respectively) while laryngeal and neck cancers made up about 7.5 % of cases with only 1.5 % of cases were hypopharyngeal. In addition, 27.1 % of cases were stage I and II, whilst 54.9 % of cases were stages III and IV (Table 1). Within the HNSCC patient cohort, 4.5 % of patients were classified as current smokers, or having quit within the past 12 months, while 47.4 % were former smokers (quit more than one year ago) and 19.5 % have never smoked (Table 1). Although we do not have all the patient information regarding alcohol consumption, most of the recruited patients were alcohol users (71 %) (Table 1).

HPV-positive HNSCC patients (*n* = 45) were on average younger than HPV-negative HNSCC patients (*n* = 88) (mean age: 60 years, SD: 10.4 years, for HPV-positive HNSCC patients and mean age: 66 years, SD: 12.6 years for HPV-negative HNSCC patients, *p* < 0.0001) (Table 1). There were significantly more men than women patients by HPV status (93.3 % men in HPV-positive HNSCC cohort; 76.1 % men in HPV-negative HNSCC cohort, *p* < 0.0001) (Table 1). The majority of HPV-negative HNSCC patients had cancers within the oral cavity (76.1 %) whereas the majority of HPV-positive HNSCC patients had cancers in the oropharynx (86.7 %) (Table 1). Compared to HPV-negative HNSCC patients, HPV-positive HNSCC patients were mostly diagnosed with stage IV tumours (29.5 and 66.7 % respectively) (Table 1). This is primarily due to the higher frequency of patients with N2 neck disease that is commonly seen in HPV-positive HNSCC [42]. Most HPV-negative and HPV-positive HNSCC patients were current (31.8 and 22.2 % respectively) and former (45.5 and 51.1 % respectively) smokers (Table 1).

### Evaluate the stability of bisulfite-converted DNA

To achieve the uniformity across all of the MSP assays carried out at different times, the stability of the bisulfite converted DNA was tested. MSPs were carried out using converted DNA on five methylated DNA tumour-suppressor genes on a weekly basis for three months. Our densitometry results showed consistency (coefficient of variation, CV of <5 %) across the three month time period when bisulfite converted DNA templates were stored at 4 °C, demonstrating the stability of the MSP reactions (data not shown). All of the MSP data used in this paper were generated within three months’ time period.

### Evaluate the specificity of MSP primers

MSP primers for individual tumour-suppressor gene were investigated using bisulfite unconverted DNA. When using bisulphite unconverted DNA, we were unable to detect any PCR amplifications further confirming the specificity of our MSP primers. In addition, as stated above, all of the five DNA methylation tumour genes investigated in this study have been extensively validated previously [6, 25, 33–35].

### Evaluate the reproducibility of MSP

Inter and intra-assay variations were carried out using randomised samples for all five methylated DNA tumour-suppressor genes. The inter- and intra-assay CVs fell within the range of 10 to 20 % for all of the studied genes. The limit of detection for our MSP assays were: 1.25 ng/μL of bisulfite-converted DNA for *RASSF1α*, 20 ng/μL of bisulfite-converted DNA for *p16* ^*INK4a*^ and *TIMP3* and 25 ng/μL of bisulfite-converted DNA for *PCQAP* respectively.

### Five individual tumour-suppressor gene DNA methylation levels in saliva collected from HNSCC patients and normal healthy controls

**Fig. 2:**
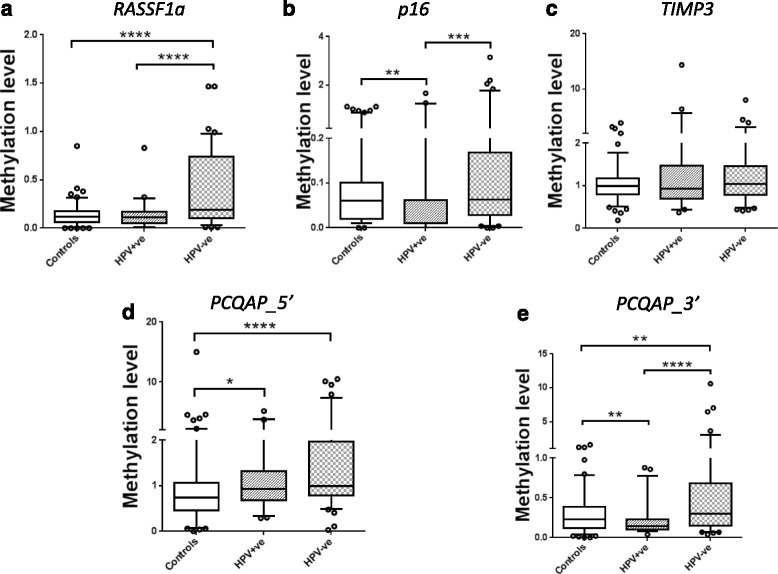
Overall DNA methylation profiles in the three groups. Whisker-box plot for the methylation signatures of **a** *RASSF1α*, **b** *p16* ^*INK4a*^, **c** *TIMP3*, **d** *PCQAP* 5′ and **e** *PCQAP* 3′ in the saliva of normal healthy controls (*n* = 122), HPV-positive (*n* = 45) and HPV-negative (*n* = 88) HNSCC patients with inter-quartile range and median shown using non-parametric Mann-Whitney’s *U*-test. Significant difference between each categories were marked with * = *p* < 0.05; ** = *p* < 0.01; *** = *p* < 0.001; **** = *p* < 0.0001, respectively

The five individual tumour-suppressor gene DNA methylation levels showed no significant association with age. DNA methylation levels were relatively higher in saliva collected from HPV-negative patients whilst lower in saliva collected from HPV-positive HNSCC patients compared with normal healthy controls (Additional file 4: Table S2). *RASSF1α*, *PCQAP 5′* and *PCQAP 3′* were significantly (*p* < 0.0001, *p* < 0.0001 and *p* < 0.005 respectively) hypermethylated in saliva collected from HPV-negative HNSCC patients whilst *p16* ^*INK4a*^, *PCQAP 5*′and *PCQAP 3′* were significantly (*p* < 0.005, *p* < 0.05 and *p* < 0.005 respectively) hypomethylated in the saliva collected from HPV-positive HNSCC patients compared with normal healthy controls (Fig. 2). Table 3 summarises the predictive accuracies for the five individual tumour-suppressor genes.

**Table 3:** The clinical performance for the individual tumour suppressor genes

HPV-status	Biomarker/Predictor	Sensitivity (%)	Specificity (%)	PPV (%)	NPV (%)	AUC	*p*-value
HPV-negative	*RASSF1a*	41	92	66	80	0.69	<0.0001*
	*p16* ^*INK4a*^	47	69	62	55	0.55	0.12
	*TIMP3*	37	82	62	62	0.56	0.10
	*PCQAP 5*′	82	46	76	55	0.70	<0.0001*
	*PCQAP 3*′	34	85	62	64	0.59	<0.005*
HPV-positive	*RASSF1a*	68	40	75	32	0.53	0.77
	*p16* ^*INK4a*^	73	67	86	48	0.69	<0.005*
	*TIMP3*	27	92	75	58	0.51	0.94
	*PCQAP 5*′	81	44	84	38	0.62	<0.05*
	*PCQAP 3*′	76	63	86	46	0.68	<0.005*

The summary of predictive accuracy of the five individual DNA methylation genes in saliva collected from normal healthy controls and HPV-negative and HPV-positive HNSCC patients using Mann-Whitney’s *U*-test and receiver operative characteristic curve. Significant difference between each category was marked with *

### Differential diagnosis of HPV-negative and HPV-positive HNSCC patients using the five tumour-suppressor gene panel

**Fig. 3:**
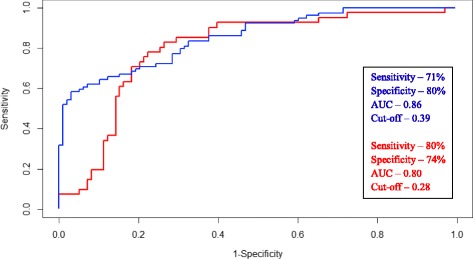
Performance of the panel in detecting HPV-negative and positive HNSCC. Carstensen’s multivariate receiver-operating characteristics curve when all of the five salivary methylation genes are combined, comparing normal healthy controls (*n* = 122) with HPV-negative HNSCC patients (*n* = 88) (*blue bar*); and normal healthy controls (*n* = 122) with HPV-positive (*n* = 45) HNSCC patients (*red bar*) respectively

The Carstensen’s multivariate receiving operating characteristic, ROC curve offers the best case scenario of the panel’s performance based on the original samples that were used in building the model (Fig. 3). With this approach, this panel performed extremely well with the area under curve (AUC) of 0.86, sensitivity of 71 % and specificity of 80 % when discriminating HPV-negative HNSCC patients from normal healthy controls; and AUC of 0.80, sensitivity of 80 % and specificity of 74 % when comparing HPV-positive HNSCC patients with normal healthy controls (Fig. 3). The data was then processed using five-fold cross-validation and bootstrap to determine the performance of this panel in a ‘most likely scenario’ with the intention of clinical translation. The results obtained suggest that the panel is more appropriate for HPV-negative HNSCC diagnosis as the sensitivity and specificity were least influenced by the enforced probability (Table 4).

**Table 4:** Validation test of the five tumour suppressor genes as a panel

	Mean	Bootstrap SD	2.50 %	97.50 %	Pval
(a) Diagnostic potential of the panel for HPV-negative HNSCC					
Sensitivity	0.67	0.14	0.38	0.94	0.16
Specificity	0.83	0.13	0.50	1.00	0.04
PPV	0.79	0.12	0.55	1.00	0.003
NPV	0.77	0.08	0.63	0.94	0
(b) Diagnostic potential of the panel for HPV-positive HNSCC					
Sensitivity	0.76	0.17	0.33	1.00	0.15
Specificity	0.67	0.14	0.35	0.90	0.16
PPV	0.53	0.12	0.33	0.78	0.74
NPV	0.87	0.08	0.70	1.00	0
(c) Diagnostic potential of the panel for HNSCC irrespective of HPV status					
Sensitivity	0.59	0.14	0.33	0.88	0.46
Specificity	0.78	0.15	0.45	1.00	0.07
PPV	0.78	0.10	0.59	1.00	0
NPV	0.62	0.08	0.50	0.8	0.04

Four main quantities commonly assessed in a diagnostic test (namely; sensitivity, specificity and positive and negative predictive value) are calculated for this panel. The table was formulated into three grouping for three different comparisons: (a) HPV-negative HNSCC patients against normal healthy controls, (b) HPV-positive HNSCC patients against normal healthy controls and lastly (c) all HNSCC patients (regardless of HPV status) against normal healthy controls. The results shown are the mean, standard deviation, 95 % confidence interval and the p value (assessed from the null hypothesis value of 0.5) for 5000 bootstrap samples, using five-fold cross-validation

### TCGA data for HNSCC tumour and normal tissues

The criteria for probes selection for individual tumour-suppressor gene are based on whether the probes are situated in the region of methylated cites amplified by MSPs. Four of our DNA methylation loci could be found in the TCGA data base and these were *RASSF1α*, *TIMP3*, *PCQAP* 5′ and *PCQAP* 3′. We were unable to locate a corresponding probe relevant to *p16* ^*INK4a*^ in the TCGA data base (Additional file 5: Figure S3). While the methylation data for *RASSF1α* from TCGA correlated with the DNA methylation levels in saliva collected from HPV-positive HNSCC patients, the overall methylation status of *TIMP3* and *PCQAP* did not vary significantly in tumour samples compared to salivary DNA methylation levels. This may be due to the differences in anatomical sites where tumours have been analysed in the TCGA data.

## Discussion

Differential DNA methylation in tumour-suppressor genes is a frequent event during human neoplasms [43]. DNA methylation plays a significant role in head and neck carcinogenesis [26, 27, 44]. In this study, we describe a five DNA methylation panel (*RASSF1α*, *p16* ^*INK4a*^, *TIMP3*, *PCQAP* 5′ and *PCQAP* 3′) that can discriminate HPV-negative and HPV-positive HNSCC patients from normal healthy control smokers and non-smokers. Significantly higher DNA methylation levels were observed in saliva collected from HPV-negative HNSCC patients compared with normal healthy controls. In contrast, a significant reduction in DNA methylation was detected in saliva collected from HPV-positive HNSCC patients compared with HPV-negative HNSCC patients. In general, DNA methylation levels were similar or lower in the saliva collected from HPV-positive HNSCC patients compared with the saliva collected from normal healthy controls. Our data corroborates previously published findings that HPV integration reduces global methylation levels [45, 46].

DNA methylation in tumour-suppressor genes is an early event in tumorigenesis; hence, it is likely to represent an ideal biomarker to evaluate early-stage tumour activities [43]. Based on the current literature, all four of the genes analysed in our study have vital roles in regulating cell proliferation either directly or indirectly [47–56]. Down regulation of *RASSF1α* was found in many cancer types including head and neck, lung, breast, prostate, ovarian, gastric, bladder and colorectal [57–64]. Promoter regions of *RASSF1α* were found to be hypermethylated in tumour tissues compared to normal tissues [57–63]. In addition, numerous studies have shown that the DNA methylation levels in saliva for *RASSF1α* mirrors actual tumour activities [6, 26, 34, 65].

*p16* ^*INK4a*^ protein expression in tumour tissue samples is a current gold stand to determine HPV status in HNSCC patients [32, 66]. This is due to the fact that while the promoter region of *p16* ^*INK4a*^ is hypermethylated in most cancer types, it was found to be significantly hypomethylated (elevated protein expression) in HPV-positive HNSCC tumour tissues as well as in saliva samples [67]. During HPV-16 integration, HPV-16 E7 binds to pRb and releases E2F which then result in rapid cellular proliferation, resulting in higher expression of *p16* ^*INK4a*^ [68]. A significant reduction in *p16* ^*INK4a*^ DNA methylation was observed in saliva from HPV-positive HNSCC patients compared with saliva from normal healthy controls, further confirming the diagnostic utility of *p16* ^*INK4a*^ protein expression in tumour tissues for determining HPV status. In addition, our findings also corroborated with previous literature, further enforcing the distinct biological and clinical features between HPV-positive and HPV-negative HNSCC patients [5, 7, 69].

*TIMP3* DNA methylation levels were higher in saliva collected from HPV-negative HNSCC patients compared to normal healthy controls. DNA promoter hypermethylation of *TIMP3* has shown to be strongly associated with HNSCC pathogenesis [70–72]. According to recent publications, DNA methylation of *TIMP3* is a robust biomarker, which can also predict HNSCC recurrences [34, 70–73]. In addition, the *TIMP3* methylation levels in tumour tissues were able to predict the formation of secondary tumours [73].

While *RASSF1α*, *p16* ^*INK4a*^ and *TIMP3* have been extensively studied as useful biomarkers to detect HNSCC, *PCQAP*/*MED15* has been identified by our group [25]. In this study, we were able to demonstrate unequivocally that the salivary DNA methylation levels of *PCQAP*/*MED15* could discriminate between normal healthy controls and HPV-negative and HPV-positive HNSCC patients. *PCQAP/MED15* encodes for a protein complex member of the transcriptional co-activator mediator family, specifically the RNA polymerase II transcriptional subunit 15 [51]. It is responsible for the transcriptional regulation of ligand-activated proteins such as the transforming growth factor betas (TGFβs) [51]. TGFβs play a role in cellular regulation, proliferation and differentiation [51]. As such, *PCQAP/MED15* may have an important role as a tumour-suppressor gene [51]. According to PubMeth database (Ghent University, Ghent, Kortrijk, Belgium), *PCQAP/MED15* is hypermethylated in over 66 % of oesophageal and 40 % of prostate cancers. This gene contains two annotated CpG islands, one at the main promoter region and another overlapping with the 14th exon (Additional file 3: Figure S2).

Standard MSP is often regarded as a qualitative analysis; it is also not informative when determining the percentage of methylation levels [74]. However, to quantify methylation bands, we used MSP coupled with densitometry software such as ImageJ. We have also made sure that the band intensities were not saturated and that all of the MSPs were in the linear range of the calibration standard. Based on the results, the MSP assay for the five individual tumour-suppressor genes has been optimised to operate in a linear range (*R* ^*2*^ > 0.8). In order to minimise inter-assay variation, two independent researchers quantified the bands and an average value was taken when the results deviated >10 %. The CV of our assays fell within the range of acceptable precision and repeatability, demonstrating that the results are reliable. In addition, the efficiency of MSP was also investigated to address nested-MSP bias.

## Conclusion

The differential DNA methylation in tumour-suppressor genes can potentially be used in identifying early-stage HPV-negative HNSCC patients as well as to classify their HPV status accordingly. This indicates that not only can this panel recognize but also categorize patients based on their salivary DNA methylation signature profiles. We’ve also used two advanced statistical methods to demonstrate the clinical relevance of this panel. Our panel was subjected to a five-fold cross-validation and bootstrap statistical analyses and was able to detect HPV-negative HNSCC with high sensitivity and specificity. This is a great clinical end point as it would mean that testing a single saliva sample with a simple DNA methylation test; one would be able to accurately discern three clinical outcomes for a patient in a non-invasive fashion. Furthermore, since the DNA is isolated from saliva, tumour-suppressor methylation signature changes are likely to have originated from tumour cells. In the future, randomised, multi-site and double-blinded studies will be a highly-informative prelude to clinical implementation of this panel to detect and discriminates HPV-positive and HPV-negative HNSCC.

## Additional files

Additional file 1: Figure S1.
*MED15/PCQAP* MSP amplicon sequence confirmation. The alignment of *MED15*/*PCQAP* MSP amplicon sequence in the NCBI Basic Local Alignment Search Tool (BLAST) database. (DOCX 218 kb)
Additional file 2: Table S1. The Cancer Genome Atlas data file. The raw data extracted from The Cancer Genome Atlas (TCGA) database, containing the methylation signature information for *RASSF1α*, *TIMP3* and *PCQAP* in HNSCC and normal tissues. (XLSX 101 kb)
Additional file 3: Figure S2. The amplification regions of individual genes with respective promoter start sites and CpG islands. A detailed map illustrating the location of the amplification region, promoter start site and CpG islands for *RASSF1α*, *p16* ^*INK4a*^, *TIMP3* and *PCQAP*. (DOCX 260 kb)
Additional file 4: Table S2. Methylation ratio data file. The raw data generated for this study. Each table contains the band intensity ratio of methylated to unmethylated MSP. The file contains the methylation ratio of *RASSF1α*, *p16* ^*INK4a*^, *TIMP3* and *PCQAP* for normal healthy controls and HPV-negative and HPV-positive HNSCC patients used in this study. (XLSX 31 kb)
Additional file 5: Figure S3. Methylation pattern in tumours and normal tissues. The methylation signature of *RASSF1α*, *TIMP3* and *PCQAP* in HNSCC and normal tissues from The Cancer Genome Atlas (TCGA) database. (DOCX 91 kb)

## Abbreviations

AUC: Area under curve
HNSCC: Head and neck squamous cell carcinoma
HPV: Human papillomavirus
IHC: Immunohistochemistry
MSP: Methylation-specific polymerase chain reaction
PAH: Princess Alexandra Hospital
ROC: Receiver operating characteristic
TCGA: The Cancer Genome Atlas
TGFβ: Transforming growth factor beta
TNM: Tumour-node-metastasis
