# Supplementary material for: Salivary DNA methylation panel to diagnose HPV-positive and HPV-negative head and neck cancers
Source: BMC Cancer. 2016 Sep 23;16:749. doi: 10.1186/s12885-016-2785-0 (PMC5034533; doi:10.1186/s12885-016-2785-0)
Supplement: Additional file 1: Figure S1. — MED15/PCQAP MSP amplicon sequence confirmation. The alignment of MED15/PCQAP MSP amplicon sequence in the NCBI Basic Local Alignment Search Tool (BLAST) database. (DOCX 218 kb) [file 12885_2016_2785_MOESM1_ESM.docx]

**Supplementary figure 1.** *MED15/PCQAP* MSP amplicon sequence confirmation.

*MED15*/*PCQAP* 3’ Forward sequence

AATGAGATTGCAGTTCTCGGTAGAGATCGATTGGCGGAGTATCGTTTTTCGGTAGAAGTTGGTTAGTTAAATGTGAGTAGTGGTCGGGGTAGGGGGTTGGATTGTGGGATTTTTTTTTTTAGCGTGGCGGGCGAGGTTAGGGTCTAATTAA


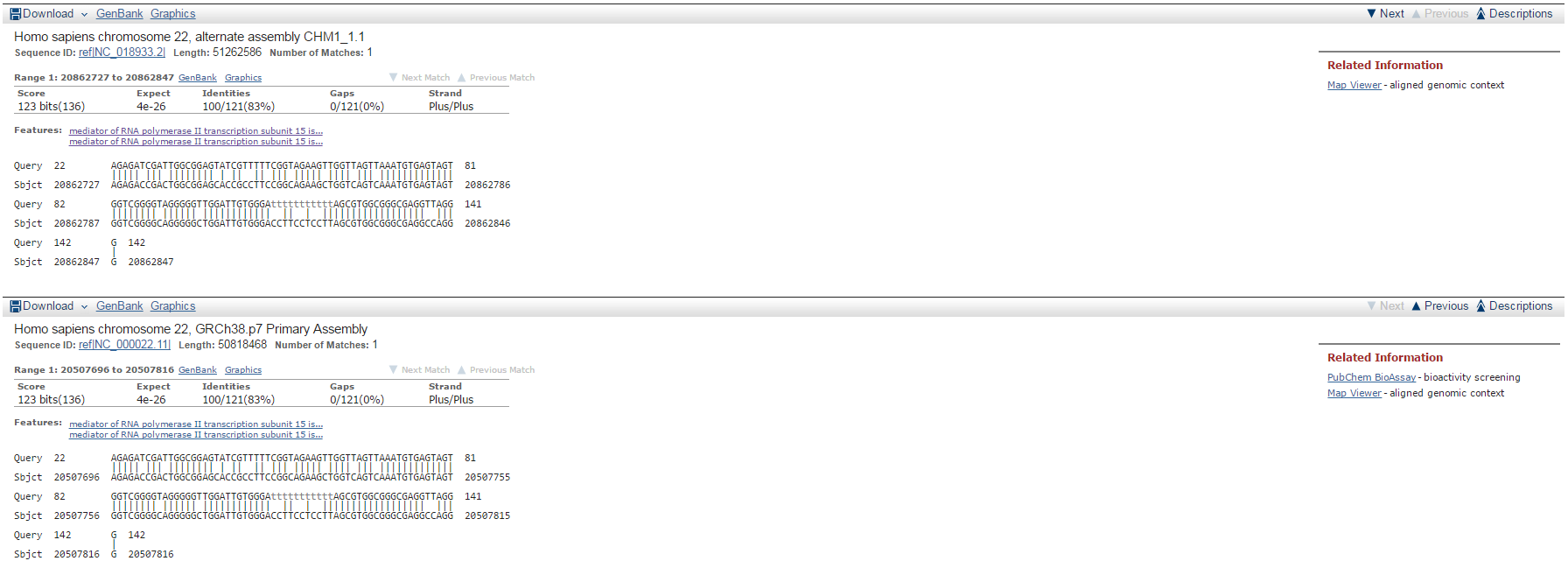
NCBI Basic Local Alignment Search Tool (BLAST) result:

*Link:*

*(http://www.ncbi.nlm.nih.gov/nucleotide/528476531?report=gbwithparts&from=20862710&to=20941712&RID=TX6ZY13G01R)*

*MED15*/*PCQAP* 5’ Forward sequence


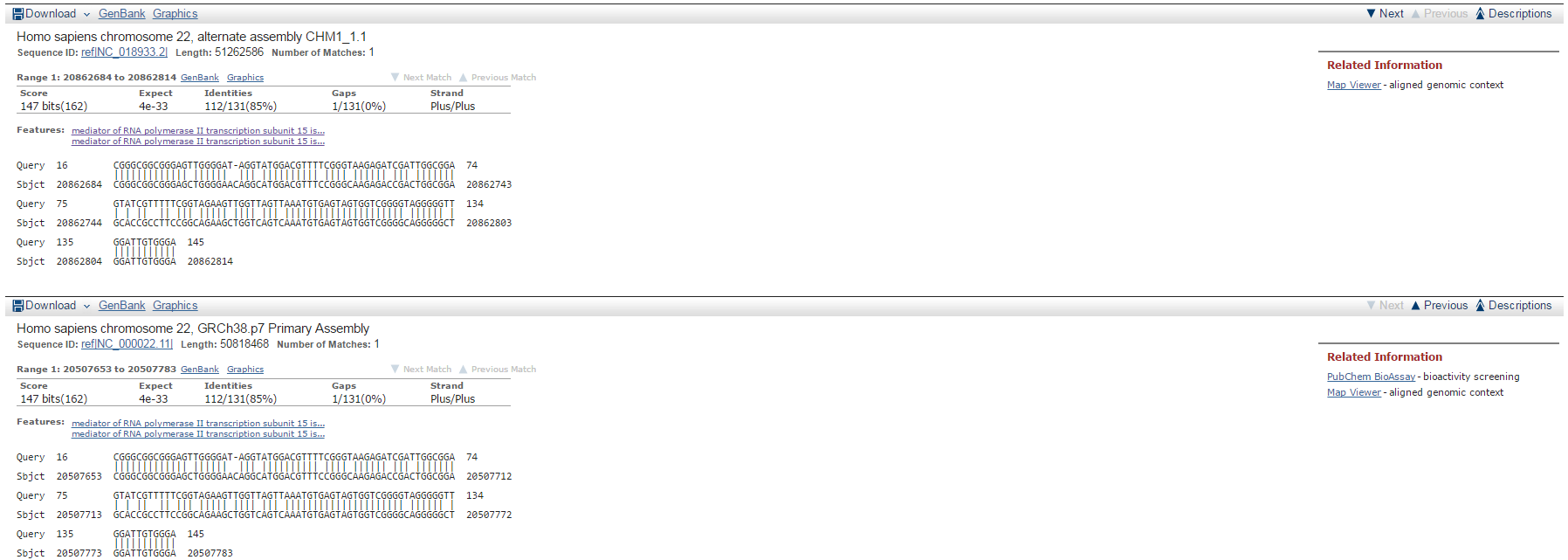
CAGGATCGTAACGGTCGGGCGGCGGGAGTTGGGGATAGGTATGGACGTTTTCGGGTAAGAGATCGATTGGCGGAGTATCGTTTTTCGGTAGAAGTTGGTTAGTTAAATGTGAGTAGTGGTCGGGGTAGGGGGTTGGATTGTGGGATTTTT

NCBI Basic Local Alignment Search Tool (BLAST) result:

*Link:*

*(http://www.ncbi.nlm.nih.gov/nucleotide/528476531?report=gbwithparts&from=20862710&to=20941712&RID=TX7TPC19014)*
