# Supplementary material for: Salivary DNA methylation panel to diagnose HPV-positive and HPV-negative head and neck cancers
Source: BMC Cancer. 2016 Sep 23;16:749. doi: 10.1186/s12885-016-2785-0 (PMC5034533; doi:10.1186/s12885-016-2785-0)

**Supplementary figure 2.** The amplification region of individual genes with respective promoter starts site and CpG islands.


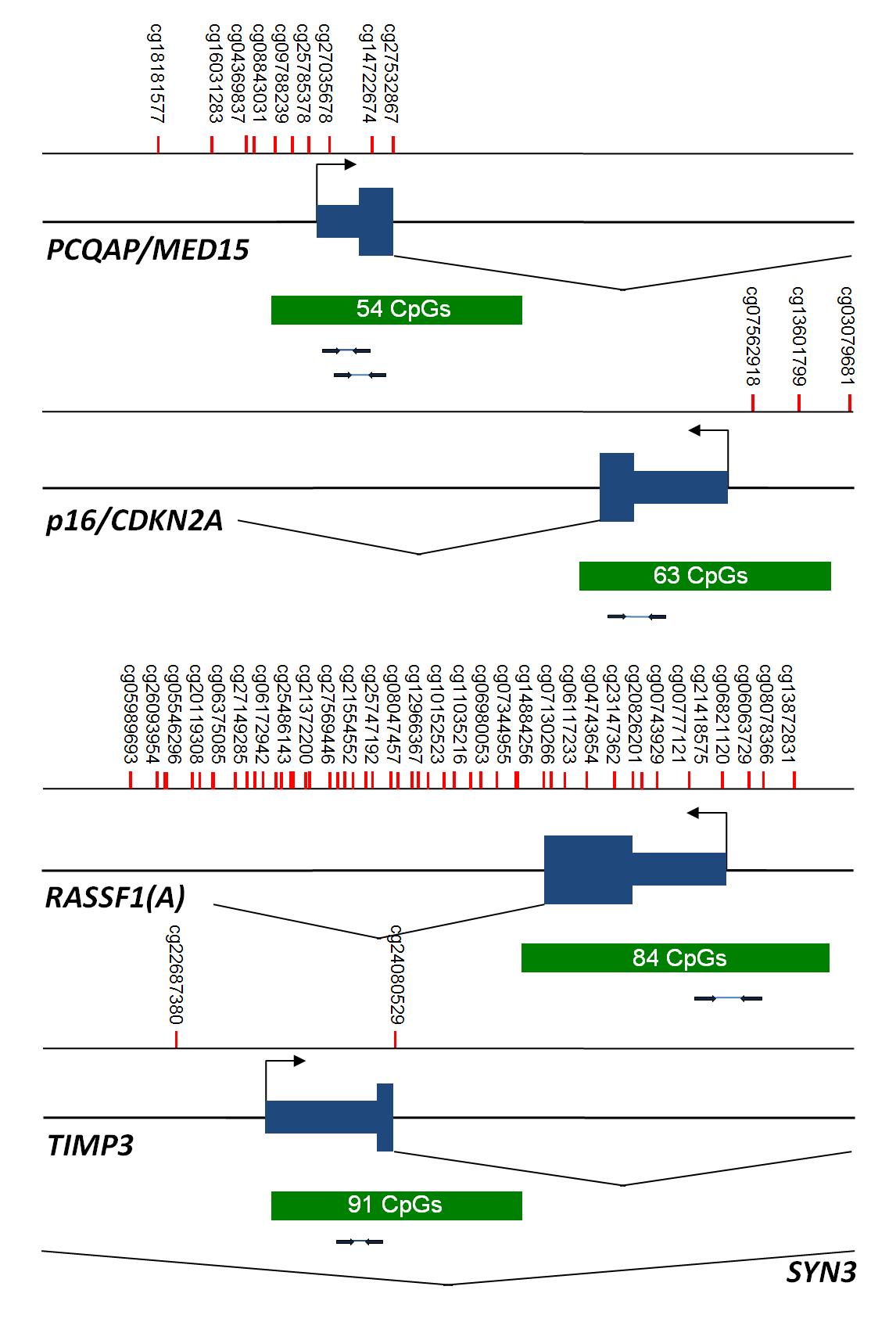

Supplement: Additional file 3: Figure S2. — The amplification regions of individual genes with respective promoter start sites and CpG islands. A detailed map illustrating the location of the amplification region, promoter start site and CpG islands for RASSF1α, p16 INK4a, TIMP3 and PCQAP. (DOCX 260 kb) [file 12885_2016_2785_MOESM3_ESM.docx]
