# Supplementary material for: Salivary DNA methylation panel to diagnose HPV-positive and HPV-negative head and neck cancers
Source: BMC Cancer. 2016 Sep 23;16:749. doi: 10.1186/s12885-016-2785-0 (PMC5034533; doi:10.1186/s12885-016-2785-0)
Supplement: Additional file 5: Figure S3. — Methylation pattern in tumours and normal tissues. The methylation signature of RASSF1α, TIMP3 and PCQAP in HNSCC and normal tissues from The Cancer Genome Atlas (TCGA) database. (DOCX 91 kb) [file 12885_2016_2785_MOESM5_ESM.docx]

**Supplementary figure 3.** Methylation pattern in tumours and normal tissues.


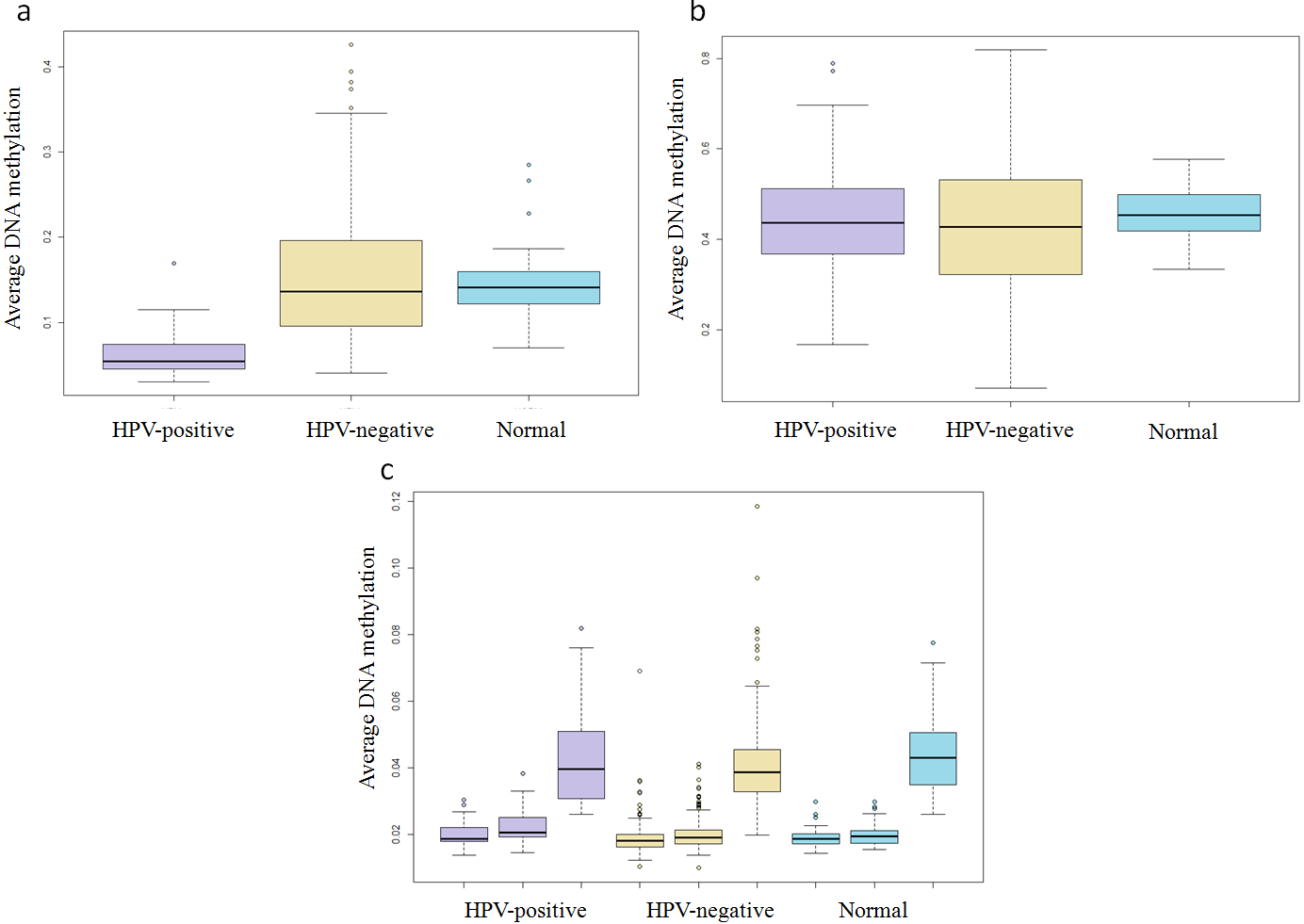


Whisker-box plot for the methylation status of (a) *RASSF1a*, (b) *TIMP3*, and (c) *PCQAP* in the tumours of HPV-negative (n=223) and HPV-positive (n=44) HNSCC and normal tissues (n=50) using information extracted from the TCGA data portal (Tang *et al*., 2013).
